# Lessons from the field: understanding the use of a youth tailored U = U tool by peer educators in Lesotho with adolescents and youth living with HIV

**DOI:** 10.1002/jia2.26267

**Published:** 2024-05-27

**Authors:** Cosima Lenz, The Committee of African Youth Advisors, Thabelang Rabaholo, Matsepo Mphafi, Felleng Samonyane, Lauren Greenberg, Angelique Thomas, Elona Toska

**Affiliations:** ^1^ Elizabeth Glaser Pediatric AIDS Foundation Medical and Scientific Affairs Washington DC USA; ^2^ Elizabeth Glaser Pediatric AIDS Foundation Maseru Lesotho; ^3^ Centre for Social Science Research University of Cape Town Cape Town South Africa; ^4^ Department of Social Policy and Intervention University of Oxford Oxford UK

**Keywords:** U = U, AYLHIV, adolescents, sub‐Saharan Africa, prevention, narrative tool

## 1 INTRODUCTION

Adolescence is defined by significant socio‐emotional changes and vulnerability. Adolescents and youth living with HIV (AYLHIV) experience worse clinical HIV outcomes—adherence, retention and viral load suppression—compared to adults [1]. Novel approaches to implement evidence‐based interventions to address their unique needs and life‐stage are needed.

Undetectable = Untransmittable (U = U) is important in the comprehensive care of AYLHIV [2, 3]. U = U is a community‐driven, evidence‐based movement embodying the message that a person living with HIV who has reached and sustained an undetectable viral load (<200 copies/ml) will not transmit HIV to a sexual partner [4, 5]. As AYLHIV navigate friendships, sexual and romantic relationships, and parenthood, U = U may be a powerful tool for safe relationships and motivation for maintaining viral suppression [6]. Limited U = U interventions exist for AYLHIV, especially in Eastern and Southern Africa, where nearly 80% of AYLHIV reside.

### 1.1 Context

Promoting U = U to address self‐stigma and accurate knowledge sharing between sexual partners has the power to shape AYLHIV's ability to live full and healthy lives [3].

Despite global support and conclusive evidence for U = U, varying levels of awareness, reception and integration in standard clinical practice remain [7]. There is a critical gap in the literature and knowledge regarding how to message and use U = U to empower AYLHIV across sub‐Saharan Africa (SSA). We embarked on creating a tool to address this gap and subsequently aimed to understand how this tool is being used and whether it is relevant to AYLHIV around U = U.

### 1.2 The U = U graphic novel

The Elizabeth Glaser Pediatric AIDS Foundation's (EGPAF) Committee of African Youth Advisors (CAYA) developed an innovative U = U tool in partnership with researchers at the University of Cape Town.

CAYA engages 15‐ to 29‐year‐old youth from 11 SSA countries. CAYA identified U = U as a priority in their 2022 prioritization survey. Members completed initial surveys on U = U knowledge and awareness, informing skill‐building sessions. CAYA previously developed cartoon‐based tools on HIV‐treatment literacy, which are routinely used in AYLHIV programming and positively received [8]. In discussions on format, CAYA preferred the tool to be visually appealing, relevant, youth‐friendly and use simple language. CAYA, therefore, decided to create a U = U graphic novel. Graphic novels and comic books can be effective in delivering clinical information to youth and can be integrated into individual/group‐based programming [9]. A graphic novel, compared to a video, can be utilized in various ways—role plays, individual/group reads, play‐acting and so on, for participatory‐based learning [9]. Paper‐based tools are useful as not all health facilities and adolescents have access to reliable technology or connectivity for standardized use of video‐based learning.

We conducted several discussions with CAYA to identify relevant U = U scenarios for youth. The three main scenarios identified included U = U in the context of romantic partnerships (heterosexual and same‐sex) and pregnant young women. CAYA reflected on characters for each scenario and developed character profiles for each story. We iteratively developed the stories and worked with local African artists for illustration.

The stories include:

1. *Lira and Obi*: A young man and woman in a new relationship navigating HIV disclosure
2. *Martha and Ibrahim*: A sero‐discordant married couple, Martha is newly pregnant
3. *Tambo and Junior*: Two young men as a couple navigating disclosure and prevention of HIV

### 1.3 Integration of the tool in Lesotho

EGPAF Lesotho implements youth‐responsive HIV services supported by youth ambassadors (YA). U = U messaging is included in national guidelines on HIV treatment and prevention. YA are trained peers who facilitate all AYLHIV peer support groups (PSGs), conduct individual counselling and support health services at adolescent corners and other facilities. Utilizing YA to implement the tool was logical as the tool was designed for use with AYLHIV in individual and group settings supported by capacitated peers. YA were introduced to the U = U graphic novel and oriented on how to integrate it into programming during a training in December 2022.

YA facilitate PSGs for different ages 10−14, 15−19, 20−24 and groups including pregnant and breastfeeding girls. Twenty‐seven YA support 238 PSGs and 75 Ariel Clubs across eight districts. YA facilitate PSGs on weekends at facilities ranging in size between 40 and 60 AY. At the end of 2023, 5300 AY were actively participating in PSGs facilitated by YA.

In August 2023, we sought to understand how YA use the tool and their feedback on its use. We developed an e‐survey with questions focused on how the tool is used, the stories used in what different settings (PSGs, individual counselling, etc.), perceptions of the usefulness and relevance, confusions raised on U = U and suggestions on improving the tool. EGPAF Lesotho staff shared an English and Sesotho survey link via email and on WhatsApp to the 27 YA. No personal identifiable data were collected, and all responses were anonymized.

### 1.4 Field insights

Twenty (74%) YA completed the survey. Eighty percent (16/20) reported having used one or more of the U = U stories. YA reported using the tool in different ways: 65% (13/20) used it in PSGs with AYLHIV; 45% (9/20) used it in one‐on‐one counselling with AYLHIV; and 35% (7/20) used it personally.

All three stories were used. Ten YA reported using Lira and Obi in putting U = U in context for sexually active young people, those in relationships and navigating disclosure. Five YA utilized the Martha and Ibrahim story to support AY navigating disclosure, address misconceptions about pregnancy, process acceptance of their status and empower pregnant girls in pregnancy peer support settings. Six YA used the Tambo and Junior story as they support all AYLHIV regardless of identity and gender and to discuss disclosure and U = U with sexually active youth.

Table 1 depicts themes on the usefulness and relevance of the tools, and clarifications raised on U = U while using the tool. YA who identified the tool as useful (*n* = 14) cited reasons including it facilitated the sharing of personal stories, it provided avenues to connect adherence with living a healthy positive life and it showcased helpful context around how to disclose. The format was described as simple to use and enjoyable. Three YA described the tools as somewhat useful indicating other life experiences may not be depicted. No YA reported the tool as not useful.

**Table 1:** Themes on usefulness, relevance and clarification raised in using the U = U graphic novel

	Themes
**Usefulness** *80% of youth ambassadors described the graphic novel tool as useful (n = 13 very useful; n = 3 somewhat useful)*	***Familiarity of stories made the information relatable and useful*** ‐ Facilitated sharing of personal stories in a group ‐ Aligned to similar experiences and situations young people encounter and providing context to face their own challenges ‐ Depicted positive relationships which serviced as giving young people hope to go into romantic relationships ‐ Have familiar images ‐ Conveying the U = U message in a simple, enjoyable manner ***Graphic novels include knowledge, information and inspirational content*** ‐ Educated young people in providing accurate information on medication, adherence and its benefits ‐ Developed understanding of reasons for and how to disclose in relationships among AYLHIV and their partners ‐ Built understanding that AYLHIV can have sex if their viral load is down and there is no chance of transmitting to their partner ‐ Shared positive and inspirational messages for pregnant adolescents ‐ Offered hope to young people in having HIV‐negative children ‐ Motivated young people to get virally suppressed ***Flexible for use with individuals, couples or groups*** ‐ One‐on‐one counselling ‐ Counselling young couples ‐ Offered relatable content for different ages in group settings during peer support groups
***Relevance*** *80% of youth ambassadors described the graphic novel tool as relevant (n = 10 as very relevant; n = 6 as somewhat relevant)*	
***Confusions and clarifications*** *40% of youth ambassadors reported questions on U = U raised while using the tool (n = 8)*	***Questions raised on U = U included on the following topics***: ‐ U = U and safe sex (*n* = 8) ‐ U = U as a part of disclosing to a sexual partner (*n* = 6) ‐ U = U and other STIs (*n* = 6) ‐ U = U applying to different kinds of sex (*n* = 5) ‐ U = U and family planning or contraception (*n* = 5) ‐ Risks of U = U (*n* = 4) ‐ U = U and pregnancy, childbirth or breastfeeding (*n* = 4) ‐ Efficacy of U = U (*n* = 6) ‐ U = U and knowing one's viral load (*n* = 4) ‐ U = U and gender/sexual identity (*n* = 2)

Many YA (*n* = 10) identified the tool as very relevant because it explained adherence and viral suppression; it motivated youth to get virally suppressed; and it mirrored similar situations young people experience. The imagery and storylines were described as relevant and relatable. Six YA found the tool somewhat relevant, noting more difficulty in use with large groups, omission of other lifestyle barriers and the need for translation into Sesotho. Eight YA shared a range of clarifications/questions raised when using the tool with AYLHIV ranging from U = U and safe sex to U = U and gender/sexual identity. YA shared the tool helped AYHLIV see life is still possible when living with HIV.

Most YA respondents indicated nothing was missing from the tool. Suggestions for improvements included translating the stories into Sesotho, implementing more training on its use, digitizing the stories and adding a story depicting a young person who thinks reaching undetectable is being cured. Other suggestions included developing accompanying demonstration tools, adding more diversity in stories including elements from rural settings, and exploring options for use in school settings.

## 2 CONCLUSIONS

The experience of implementing this tool reveals the practicality and promise of a narrative, youth‐friendly graphic novel tool on a topic like U = U.

## COMPETING INTERESTS

The authors declare no competing interests.

## AUTHORS’ CONTRIBUTIONS

CL, TR, ET, AT, MM and CAYA conceptualized the project. Data tools were designed by CL, TR and MM with inputs from CAYA, ET and AT. Data collection was facilitated by TR and MM. Data analysis was led by CL. The draft was jointly constructed by all authors and reviewed by all authors.

## FUNDING

ET/AT were funded by the Fogarty International Center, National Institute on Mental Health, National Institutes of Health [K43TW011434] and UKRI GCRF Accelerating Achievement for Africa's Adolescents (Accelerate) Hub (ES/S008101/1).

## DISCLAIMER

The content is solely the responsibility of the authors and does not represent the official views of the National Institutes of Health.

## Data Availability

Data sharing is not applicable to this article as no datasets were generated or analysed during the current study.
